# Die Genetik von Vorhofflimmern – auf dem Weg in die Präzisionsmedizin

**DOI:** 10.1007/s00399-023-00974-z

**Published:** 2023-11-06

**Authors:** Shinwan Kany, Renate B. Schnabel

**Affiliations:** 1 Klinik für Kardiologie, Universitäres Herz- und Gefäßzentrum Hamburg-Eppendorf, Universitätsklinikum Hamburg-Eppendorf, Martinistraße 52, 20251 Hamburg, Deutschland; 2 Standort Hamburg/Kiel/Lübeck, Deutsches Zentrum für Herz-Kreislauf-Forschung (DZHK), Hamburg, Deutschland; 3 Cardiovascular Disease Initiative, Broad Institute of MIT and Harvard, Cambridge, MA USA

**Keywords:** Herzrhythmusstörungen, Genetik, Genomweite Assoziationsstudien, Seltene Genetische Varianten, Polygenetische Risikoscores für Vorhofflimmern, Cardiac arrhythmias, Genetics, Genome-wide association studies, Rare variants, Polygenic risk scores

## Abstract

Vorhofflimmern (VHF) ist eine verbreitete Herzkrankheit mit komplexer genetischer Grundlage. Trotz der Fortschritte in der Behandlung bleibt die Sterblichkeit bei VHF-Patienten hoch. Diese Übersichtsarbeit diskutiert die genetische Basis von VHF und ihre Auswirkungen auf Diagnose und Therapie. Während seit Langem eine hereditäre Komponente bei VHF bekannt war, wurden die ersten mit VHF assoziierten Gene in den frühen 2000er Jahren identifiziert. Mit Hilfe von genomweiten Assoziationsstudien (GWAS) wurden weitere Gene und zahlreiche genetische Varianten, die mit VHF in Verbindung stehen, identifiziert. Diese Studien haben fast 140 verschiedene, mit VHF assoziierte Regionen in der DNA aufgezeigt. Neben häufigen Varianten wurden auch seltene Varianten mit großen Auswirkungen identifiziert. Die Integration dieser genetischen Erkenntnisse in die klinische Praxis verspricht, die Diagnose und Behandlung von VHF zu verbessern und uns der Präzisionsmedizin näherzubringen. Es bleiben jedoch viele Herausforderungen, insbesondere besteht eine Diskrepanz für genetische Daten von Menschen mit nichteuropäischer Abstammung und an genetischen Analysen des therapeutischen Ansprechens.

## Abstract (en)

Atrial fibrillation (AF) is the most common cardiac arrhythmia and has complex genetic underpinnings. Despite advancements in treatment, mortality of AF patients remains high. This review discusses the genetic basis of AF and its implications for diagnosis and therapy. Although AF pathology has long been known to include a hereditary component, the first genes associated with AF were not identified until the early 2000s. Subsequent research with genome-wide association studies (GWAS) has implicated other genes and numerous genetic variants in AF. These studies have revealed nearly 140 different regions in the DNA with genome-wide significance associated with AF. In addition to common variants, rare variants with large effects have also been identified. The integration of these genetic findings into clinical practice holds promise for improving AF diagnosis and treatment, moving us closer to precision medicine. However, challenges remain, including the need for more diverse genetic data of non-European ancestry and improved genetic analyses of responses to AF therapy.

Im Jahr 2019 waren weltweit über 60 Mio. Menschen von Vorhofflimmern (VHF) betroffen [1]. Bei Menschen mit europäischer Abstammung liegt die Wahrscheinlichkeit, VHF zu entwickeln, bei 1 zu 3 für Männer und 1 zu 5 für Frauen [2]. Selbst bei optimaler Therapie ist die Sterblichkeit von Menschen mit VHF mehr als dreimal so hoch wie bei Menschen ohne VHF. Zudem erhöht VHF das Risiko für Herzinsuffizienz, akutes Koronarsyndrom und Schlaganfall, sowohl bei Männern als auch bei Frauen [2]. Aktuelle Daten zeigen, dass VHF die wahrscheinliche Ursache für 12–14 % aller Schlaganfälle ist, wobei eine hohe Dunkelziffer angenommen wird [3].

Die meisten Patient:innen mit VHF erhalten orale Antikoagulanzien zur Senkung des Schlaganfallrisikos [4]. Für die Symptombehandlung stehen medikamentöse Optionen der Frequenz- und Rhythmuskontrolle zur Verfügung. Darüber hinaus besteht mit der Katheterablation eine interventionelle Option für die Rhythmuskontrolle. Obwohl die Ablation zur Rhythmuskontrolle effektiver als Medikamente ist, bleiben Antiarrhythmika ein wichtiger Bestandteil der Therapie [5]. Der derzeitige Ansatz bei der Auswahl der medikamentösen Rhythmustherapie zielt hauptsächlich auf die Minimierung von Nebenwirkungen. Die hohe Variabilität in der Wirksamkeit aller verfügbaren Therapien ist auf die heterogene Genese von VHF zurückzuführen. Die Vererbbarkeit von VHF anhand von sog. „single nucleotide polymorphism“ (SNP) wird aktuell auf ca. 22 % geschätzt [6].

Seit der ersten Beschreibung von familiärem VHF vor über sieben Jahrzehnten [7] wurden große Fortschritte bei der Erforschung der genetischen Architektur von VHF gemacht. Obwohl genetische Ansätze gezeigt haben, dass das Risiko für VHF und die Effektivität von Therapien teilweise genetisch bedingt sind, ist der Einfluss dieser Erkenntnisse auf die Patientenversorgung noch begrenzt. In dieser Übersichtsarbeit wird die genetische Basis von VHF erläutert und die Applikation auf Diagnostik und Therapie diskutiert.

## Genetische Basis von Vorhofflimmern

Eines der ersten Gene, die mit VHF assoziiert wurden, war *KCNQ1* mit einer Gain-of-function-Mutation in einer chinesischen Familie im Jahr 2003 [8]. Weitere Gene, die durch die Untersuchung von Familien mit Anhäufung von VHF gefunden wurden, waren u. a. *SCN5A*, das Natriumkanäle kodiert, sowie *NPPA*, welches das atriale natriuretische Peptid kodiert [9, 10]. Diese aufwändigen Sequenzierungsstudien von Familien mit VHF wurden durch genomweite Assoziationsstudien (GWAS) ergänzt. Dabei werden hypothesenfrei Personen mit einem Merkmal (wie VHF) mit Personen verglichen, die das Merkmal nicht besitzen (kein VHF). In der Regressionsanalyse wurden dafür die SNPs verwendet, die auf Genotypisierungs-Arrays Mitte der 2000er erstmals genomweit zur Verfügung standen. Diese Arrays enthalten häufige Varianten („common variants“), die in der Population verbreitet vorkommen, jedoch i. d. R. geringe Effektstärken haben. Seltene Varianten („rare variants“) kommen in der Bevölkerung – wie der Name sagt – seltener vor, üben aber häufig größere Effekte aus. Der genetische Anteil komplexer Erkrankungen wie VHF ist oftmals durch den kumulativen Effekt von „common variants“ auf Populationsebene erklärbar.

### Genomweite Assoziationsstudien

Die erste große GWAS-Studie zu VHF wurde im Jahr 2007 durch die isländische deCode-Gruppe veröffentlicht. Sie umfasste 500 VHF-Probanden und 4476 Kontrollprobanden und identifizierte 2 Varianten im 4q25-Locus nahe *PITX2* [11]. Dieses Gen kodiert einen Transkriptionsfaktor, der für die Kardiogenese und Ausbildung der Rechts-Links-Differenzierung kardialer Strukturen essenziell ist [12]. Die Nutzung von GWAS wurde konsequent weiterverfolgt und führte zur Entdeckung zahlreicher weiterer Gene und genomischer Bereiche, die in Verbindung mit VHF stehen. Die aktuellen GWAS für VHF analysierten über 8 Mio. Varianten bei mehr als 500.000 Probanden, von denen über 65.000 VHF hatten [13, 14]. Dabei konnten fast 140 verschiedene Regionen in der DNA identifiziert werden, die mit genomweiter Signifikanz (*p* < 5 × 10^−8^) eine Assoziation mit VHF aufweisen. Mit funktionalen Untersuchungen und Sequenzierungsstudien konnten u. a. Gene für Transkriptionsfaktoren (*PITX2, ZFHX3, TBX5*), Ionenkanäle (*KCNN2, SCN10A, HCN4, KCNH2*), Strukturen des Zytoskeletts (*MYH6, MYH7, PKP2*) und Calcium-Signaling (*CAMK2D, PLN*) gefunden werden [15].

### Monogenetische Einflüsse auf Vorhofflimmern

Neben der Rolle von „common variants“ für das gesamte genetische Risiko von Vorhofflimmern (VHF) wurden auch „rare variants“ mit großem Effekt in zahlreichen Studien identifiziert. In einer Analyse mit Ganzgenomsequenzierung durch die isländische deCode-Gruppe aus dem Jahr 2017 wurden beispielsweise Loss-of-function-Mutationen in Genen nachgewiesen, die für strukturelle Proteine kodieren, wie *PLEC* und *MYH6* [16].

Besondere Bedeutung kommt hierbei dem *TTN*-Gen zu, das für Titin kodiert, dem größten Protein im menschlichen Körper. In einer ersten umfangreichen Analyse mittels Ganzgenomsequenzierung von etwa 2800 Probanden mit früh einsetzendem VHF (unter 66 Jahren) und etwa 5000 Kontrollprobanden berichteten Choi und Kollegen über eine Prävalenz von mindestens einer Loss-of-function-Mutation bei 2,1 % aller VHF-Probanden [17]. Dabei wurde eine größere Prävalenz von *TTN*-Loss-of-Function-Varianten je jünger die Patienten waren beobachtet. So wurden bei Probanden mit einer VHF-Diagnose unter 30 Jahren *TTN-*Loss-of-function-Varianten in 6,5 % festgestellt [17].

In einer Analyse der UK Biobank konnte die gleiche Arbeitsgruppe in ca. 44.000 Probanden mit Ganzexomsequenzierung zeigen, dass sowohl polygene als auch monogene Einflüsse das Risiko von VHF beeinflussen. Die obersten 0,44 % des polygenetischen Risikos hatten eine VHF-Prävalenz von ca. 9 %. Dahingehend hatten 0,44 % der Probanden eine *TTN-*Loss-of-function-Variante, aber eine VHF-Prävalenz von 14 % [18]. Es zeigte sich, dass ein hoher polygenetischer Score auch mit einem erhöhten VHF-Risiko bei Trägern von *TTN-*’Loss-of-function-Varianten verbunden war. Eine Studie mit Probanden aus 24 Familien mit Anhäufung von VHF berichtete eine Prävalenz von „*TTN *truncating variants“ von 16,7 % und einem medianen Erkrankungsalter dieser Träger von 26 Jahren [19].

Seitdem wurden weitere seltene Varianten für *LMNA* (kodiert für Lamin), *PRRX1* oder *PKP2* durch Sequenzierungsstudien mit VHF assoziiert [20, 21]. Dabei ist zu beachten, dass diese Varianten nicht nur das Risiko für VHF beeinflussen, sondern auch Gene sind, die mit Kardiomyopathie in Verbindung stehen.

## Diagnostische Bedeutung von genomischen Methoden

### Prädiktion von Vorhofflimmern mit polygenetischen Scores

Die Entwicklung großer genetischer Datensätze und GWAS hat zur Entstehung der Technik der polygenetischen Risikoscores (PRS) geführt. Diese Methode nutzt die additiven Effekte von häufigen Varianten, um das Risiko für VHF zu prognostizieren. Dabei werden ausgewählte SNPs in gewichteten Summen zusammengefasst. Frühe PRS-Ansätze setzten auf die Pruning-and-Thresholding-Methode [22]. Hierbei werden SNPs mit genomweiter Signifikanz ausgewählt und mittels Linkage Disequilibrium korrelierende SNPs herausgefiltert. Aktuelle Methoden verwenden Bayesianische Regressionsmodelle, die Millionen von Varianten für einen Score gewichten. Khera und Kollegen konnten zeigen, dass ein aus der UK Biobank konstruierter PRS das Risiko für VHF signifikant stratifizieren kann. Die 6,1 % der Probanden mit dem höchsten PRS hatten ein dreifach erhöhtes Risiko für eine zukünftige Diagnose von VHF [23]. Dieser Score wurde auch in einer Studie mit mehr als 36.000 Probanden der randomisierten kontrollierten Studien der TIMI-Gruppe getestet. Hierbei war eine Standardabweichung des PRS über einen Follow-up-Zeitraum von 2,3 Jahren mit einem um 40 % erhöhten Risiko für zukünftiges VHF assoziiert [24]. Der PRS in der TIMI-Studie zeigte additive Effekte zum klinischen CHARGE-AF-Risikoscore sowie zur Nutzung von NT-proBNP als Risikomarker. In einer Studie der FinnGen-Arbeitsgruppe wurde gezeigt, dass die obersten 2,5 % eines PRS für VHF ein Lebenszeitrisiko von über 60 % für eine zukünftige VHF-Diagnose haben, verglichen mit 24,4 % für Menschen mit durchschnittlichem PRS [25]. Die gleichen Probanden erhielten eine VHF-Diagnose etwa 7 Jahre vor Personen mit durchschnittlichem PRS. Bezüglich der Vorhersage von Rezidiven nach Katheterablation ist die Datenlage gemischt. Eine Multicenterstudie untersuchte 4267 Patienten, die sich einer Katheterablation für VHF unterzogen haben und genotypisiert wurden. Es zeigte sich keine signifikante Assoziation zwischen genetischem Risiko und der Rekurrenz von VHF nach Ablation [26]. Im Gegensatz dazu zeigte sich in der genetischen Substudie der randomisierten kontrollierten EAST-AFNET4-Studie von früher Rhythmuskontrolle im Vergleich zur üblichen Versorgung eine statistisch signifikante Assoziation von PRS mit dem Wiederauftreten von VHF, wenn auch mit geringer Effektstärke (Hazard-Ratio [HR] 1,08; 95 % Konfidenzintervall [KI] 1,0−1,16; *p* = 0,047; [27]). In Tab. 1 werden selektierte Ergebnisse aus Studien gezeigt, die PRS in der Diagnostik oder Risikoeinschätzung von VHF verwendet haben.

**Table 1:** 

Studie	Fragestellung	HR/OR und 95 % KI	Studienpopulation
[23]	Prädiktion von neu diagnostiziertem VHF	OR 2,43 (2,29–2,59) der Top 20 % vs. restliche 80 %	288.978 Probanden der UK Biobank, populationsbasiert
[24]	Prädiktion von neu diagnostiziertem VHF	HR 1,40 (1,32–1,49) pro SD vom PRS	36.662 Patienten aus TIMI-Studien
[28]	Prädiktion von postoperativem VHF	OR 1,63 pro SD vom PRS	1047 Patienten nach kardiochirurgischer Operation
[25]	Prädiktion von Alter bei VHF Diagnose	Top 2,5 % des PRS wurden 6,64 Jahre vor restlichen 97,5 % des PRS diagnostiziert	135.300 Probanden der FinnGen-Studie, populationsbasiert
[26]	Rezidiv von VHF nach Katheterablation	HR 1,06 (0,98–1,15) von Top 20 % vs. restliche 80 %	4266 Patienten in retrospektiver Multicenterstudie
[27]	Rezidiv von VHF nach früher Rhythmuskontrolle	HR 1,08 (1,0–1,16) pro SD von PRS	1567 Patienten mit Vorhofflimmern der EAST-AFNET4-Studie

*HR* Hazard-Ratio, *KI* Konfidenzintervall, *OR* Odds-Ratio, *SD* Standardabweichung, *PRS* polygenetischer Risikoscore, *VHF* Vorhofflimmern

### Testung von seltenen Varianten bei frühem VHF

Obwohl es für Erkrankungen wie die dilatative Kardiomyopathie spezifische Empfehlungen für genetische Tests in den Leitlinien gibt, fehlen solche Empfehlungen in den aktuellen ESC Leitlinien zum VHF. Ein Konsensus-Dokument der Europäischen, Amerikanischen und Lateinamerikanischen Gesellschaften für Rhythmologie lieferte vor Kurzem eine Empfehlung zur genetischen Testung bei VHF ab [29]. Die Empfehlung beschränkte sich hierbei auf familiäres VHF bei jungen Patienten (< 60 Jahre) und spezifische Gene (*SCN5A, KCNQ1, MYL4* und „truncating“ *TTN-*Varianten).

Im klinischen Alltag werden für genetische Tests in der Regel Gen-Panels verwendet, die Varianten testen, die als „pathogen“ oder „wahrscheinlich pathogen“ eingestuft werden. Diese Gen-Panels sind nicht standardisiert und weichen von Anbieter zu Anbieter ab. Dabei ist zu beachten, dass zahlreiche Gene, die mit VHF in Zusammenhang stehen, ebenso als pathogen für Kardiomyopathien gelten. Die Auswahl und Definition der konkreten Varianten in einem Panel sind daher maßgeblich bei der Interpretation der Ergebnisse.

In einer Studie mit fast 1300 Patient:innen mit früh einsetzendem VHF (unter 66 Lebensjahren) wurde ein Panel von Arrhythmie- und Kardiomyopathie-Genvarianten getestet. Die Varianten wurden hierbei nach strengen Kriterien des American College of Medical Genetics and Genomics (ACMG) ausgewählt [30].

Bei 10,1 % der Patient:innen wurde eine krankheitsassoziierte Variante (am häufigsten *TTN*) und bei 62,8 % mindestens eine Variante mit unklarer Signifikanz gefunden. Die Prävalenz von pathogenen oder wahrscheinlich pathogenen Varianten stieg auf 16 % bei Patient:innen, bei denen Diagnose VHF vor ihrem 30. Lebensjahr gestellt worden war [31]. In einer Folgestudie konnte auch eine prognostische Bedeutung gezeigt werden. Patient:innen mit früh einsetzendem VHF und einer krankheitsassoziierten Variante hatten bei einem Follow-up von fast 10 Jahren ein um 50 % erhöhtes Mortalitätsrisiko [32].

In einer kleineren Studie zu früh einsetzendem VHF (durchschnittliches Alter etwa 27 Jahre) wurde bei 6 von 25 Patient:innen (24 %) eine pathogene oder wahrscheinlich pathogene Variante (5 davon *TTN*) gefunden. Die restlichen Patient:innen hatten bis auf eine Ausnahme seltene Varianten mit unklarer Signifikanz. Insgesamt wurden 11 Patient:innen nach unauffälligem transthorakalem Echo mittels Magnetresonanztomographie (MRT) untersucht, wobei 8 Patient:innen Anzeichen einer Kardiomyopathie aufwiesen [33]. Zusammengefasst zeigt sich bei früh einsetzendem VHF eine diagnostische Wertigkeit von genetischen Tests (etwa 16 % mit pathogenen Varianten), die vergleichbar mit nichtfamiliären Formen der dilatativen Kardiomyopathie (im Durchschnitt 19 %) ist [34, 35].

### Klinische Szenarien für genetische Testung

**Figure 1:**
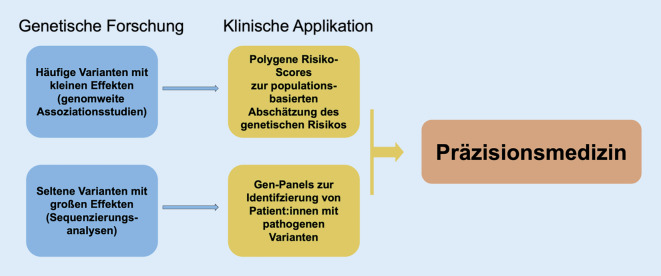

Obwohl PRS für die Diagnose und Therapie von VHF noch Gegenstand aktueller Forschung sind, können wir bereits konkrete Szenarien identifizieren, in denen Patienten von der Sequenzierung oder Panel-Testung seltener Varianten profitieren könnten (Abb. 1). Neben dem hohen diagnostischen Wert bei früh einsetzendem VHF sind viele der bekannten Varianten für VHF auch sog. *sekundäre Befunde*, wie sie vom ACMG definiert werden [36]. Diese beinhalten Varianten, die potenziell therapierelevant sind (wie *TTN, PKP2, LMNA* und andere) und deren Meldung unabhängig von der eigentlichen Indikation erfolgen sollte, wie das aktuelle Update von 2021 ausführt [37]. Die Identifizierung genetischer Varianten bei Patienten mit früh einsetzendem VHF kann daher unmittelbare Konsequenzen haben, wie z. B. die Notwendigkeit einer erweiterten Bildgebung, aber auch die Beratung und präventive Maßnahmen bei nahen Verwandten. Während es keine einheitliche Definition von frühem VHF gibt, scheint ein Alter von 45 Jahren bei einer erwarteten diagnostischen Prävalenz von 10 % an seltenen Varianten in kommerziellen Gen-Panels eine angemessene Abgrenzung zu sein [31].

Konkret sollte eine Testung von Patient:innen in Betracht gezogen werden bei:

- sehr frühem VHF (unter 45 Lebensjahren),
- familiärer Häufung von VHF vor dem 65. Lebensjahr,
- frühem VHF und Anzeichen einer nichtischämischen strukturellen Herzerkrankung,
- frühem VHF und Anzeichen von monogenetischen Defekten, wie z. B. Reizleitungsstörungen (*LMNA*) oder kurzer QT-Zeit (*KCNQ1*),
- frühem VHF und ventrikulären Arrhythmien.

## Aktuelle Entwicklungen und Ausblick

Die Genetik des VHF wird weiterhin intensiv erforscht. Neue Metaanalysen von GWAS mit doppelt bis dreifach größeren Kohorten sind derzeit beim AFGen-Konsortium in Arbeit. Dieses internationale Forschungskonsortium hat sich der genetischen Aufarbeitung von VHF verschrieben. Mit diesen neuen Daten werden weitere Einblicke in die genetische Grundlage des VHF möglich. Gleichzeitig bilden diese Daten die Grundlage für die Entwicklung neuer PRS, die das polygenetische Risiko einer Person noch effektiver quantifizieren können. Darüber hinaus haben viele Biobanken, wie die UK Biobank oder die „All of Us“-Kohorte in den USA, zunehmend Daten aus der Ganzgenomsequenzierung veröffentlicht, die auch das monogenetische Verständnis für VHF in naher Zukunft erweitern werden. Es gibt jedoch noch einige Einschränkungen, die berücksichtigt werden müssen. So stammen die meisten genetischen Daten von Menschen europäischer Herkunft, was dazu führt, dass PRS in anderen Populationen, wie Afroamerikanern, weniger aussagekräftig sind. Die Sammlung von genetischem Material von Minderheiten und diversen Populationen wurde als eines der dringendsten Ziele der aktuellen Genomforschung identifiziert. Obwohl die in Tab. 1 aufgeführten Studien verschiedene Szenarien untersuchen, wurden alle PRS auf der Basis von GWAS entwickelt, die das Risiko einer VHF-Diagnose in Biobanken abschätzen. Es ist zu erwarten, dass PRS, die auf der Basis von GWAS zur Therapieantwort auf eine Rhythmuskontrolle entwickelt wurden, eine bessere Vorhersagekraft liefern werden. Durch bessere und umfänglichere Phänotypisierung wird ermöglicht, GWAS für spezifische Fragestellung durchzuführen, die dann wiederum Werkzeuge für klinische Applikationen liefern werden. Neben einer präziseren Risikostratifizierung kann eine strukturierte genetischte Testung von „rare variants“ (Tab. 2) potenzielle Auswirkungen auf Therapieentscheidungen mit sich bringen, wie z. B. *LMNA* und frühe Implantation eines implantierbaren Kardioverter-Defibrillators (ICD).

**Table 2:** 

Gen	Protein	Studie
*LMNA*	Lamin A/C	[20]
*TTN*	Titin	[17]
*PKP2*	Plakophilin‑2	[38]
*SCN5A*	Sodium channel protein type 5 subunit alpha	[39]
*MYBPC3*	Myosin-binding protein C, cardiac-type	[40]
*KCNQ1*	Potassium voltage-gated channel subfamily KQT member 1	[8]

## Diskussion

Die Genetik des VHF hat in den letzten Jahren erhebliche Fortschritte gemacht. Die Kombination von genomweiten Assoziationsstudien, polygenen Risikoscores und der Untersuchung von seltenen Genvarianten hat zu einem detaillierteren Verständnis der genetischen Architektur dieser komplexen Erkrankung geführt. Diese genetischen Erkenntnisse haben das Potenzial, die Diagnostik und Therapie von VHF erheblich zu verbessern und uns einen Schritt näher an die Realisierung der Präzisionsmedizin zu bringen.

Trotz der Fortschritte gibt es noch viele Herausforderungen zu bewältigen. Dazu gehört die Notwendigkeit, genetische Daten aus diversen Populationen zu verstehen, um allgemeingültige Aussagen für alle Patient:innen treffen zu können. Darüber hinaus müssen wir die genetischen Analysen der Therapieantwort weiterentwickeln, um spezifischere Vorhersagen zu ermöglichen.

Insgesamt zeigt die Genetik von VHF, dass wir uns auf einem spannenden Weg in Richtung Präzisionsmedizin befinden. Mit weiterer Forschung und der Integration genetischer Daten in die klinische Praxis können wir hoffen, die Versorgung von Patienten mit VHF in der Zukunft erheblich zu verbessern.

## Fazit für die Praxis

- Die Integration genetischer Daten in die Diagnose und Behandlung von Vorhofflimmern (VHF) kann die Patientenversorgung verbessern.
- Polygene Risikoscores können zur Risikostratifizierung und Vorhersage von VHF eingesetzt werden.
- Seltene Varianten, insbesondere bei frühem VHF, können wichtige diagnostische und prognostische Informationen liefern.
- Das Verständnis der genetischen Architektur von diversen Populationen ist notwendig, um die Anwendbarkeit genetischer Erkenntnisse zu verbessern und zu verallgemeinern.
- Die Entwicklung von genetischen Analysen zu Diagnostik und Therapie wird in Zukunft zu spezifischeren Vorhersagen führen und zur Präzisionsmedizin bei VHF beitragen.

## References

[1] Dong X-J, Wang B-B, Hou F-F (2023). Global burden of atrial fibrillation/atrial flutter and its attributable risk factors from 1990 to 2019. Europace.

[2] Magnussen C, Niiranen TJ, Ojeda FM (2017). Sex differences and similarities in atrial fibrillation epidemiology, risk factors, and mortality in community cohorts. Circulation.

[3] Bernstein RA, Kamel H, Granger CB, Piccini JP (2021) … monitoring vs usual care on detection of atrial fibrillation in patients with stroke attributed to large-or small-vessel disease: the STROKE-AF randomized clinical trial. JAMA 10.1001/jama.2021.6470 PMC8170544 34061145

[4] Menichelli D, Del Sole F, Di Rocco A (2021). Real-world safety and efficacy of direct oral anticoagulants in atrial fibrillation: a systematic review and meta-analysis of 605 771 patients. Eur Heart J Cardiovasc Pharmacother.

[5] Andrade JG, Deyell MW, Macle L (2023). Progression of atrial fibrillation after cryoablation or drug therapy. N Engl J Med.

[6] Weng L-C, Choi SH, Klarin D (2017). Heritability of atrial fibrillation. Circ Cardiovasc Genet.

[7] Wolff L (1943). Familial auricular fibrillation. N Engl J Med.

[8] Chen Y-H, Xu S-J, Bendahhou S (2003). KCNQ1 gain-of-function mutation in familial atrial fibrillation. Science.

[9] Hodgson-Zingman DM, Karst ML, Zingman LV (2008). Atrial natriuretic peptide frameshift mutation in familial atrial fibrillation. N Engl J Med.

[10] Makiyama T, Akao M, Shizuta S (2008). A novel SCN5A gain-of-function mutation M1875T associated with familial atrial fibrillation. J Am Coll Cardiol.

[11] Gudbjartsson DF, Arnar DO, Helgadottir A (2007). Variants conferring risk of atrial fibrillation on chromosome 4q25. Nature.

[12] Syeda F, Kirchhof P, Fabritz L (2017). PITX2-dependent gene regulation in atrial fibrillation and rhythm control. J Physiol.

[13] Roselli C, Chaffin MD, Weng L-C (2018). Multi-ethnic genome-wide association study for atrial fibrillation. Nat Genet.

[14] Nielsen JB, Thorolfsdottir RB, Fritsche LG (2018). Biobank-driven genomic discovery yields new insight into atrial fibrillation biology. Nat Genet.

[15] Roselli C, Rienstra M, Ellinor PT (2020). Genetics of atrial fibrillation in 2020: GWAS, genome sequencing, polygenic risk, and beyond. Circ Res.

[16] Thorolfsdottir RB, Sveinbjornsson G, Sulem P (2017). A Missense variant in PLEC increases risk of atrial fibrillation. J Am Coll Cardiol.

[17] Choi SH, Weng L-C, Roselli C (2018). Association between titin loss-of-function variants and early-onset atrial fibrillation. JAMA.

[18] Choi SH, Jurgens SJ, Weng L-C (2020). Monogenic and polygenic contributions to atrial fibrillation risk: results from a national biobank. Circ Res.

[19] Ahlberg G, Refsgaard L, Lundegaard PR (2018). Rare truncating variants in the sarcomeric protein titin associate with familial and early-onset atrial fibrillation. Nat Commun.

[20] Lazarte J, Jurgens SJ, Choi SH (2022). LMNA variants and risk of adult-onset cardiac disease. J Am Coll Cardiol.

[21] Guo X, Qiu X, Wang J (2021). PRRX1 loss-of-function mutations underlying familial atrial fibrillation. J Am Heart Assoc.

[22] Choi SW, Mak TS-H, O’Reilly PF (2020). Tutorial: a guide to performing polygenic risk score analyses. Nat Protoc.

[23] Khera AV, Chaffin M, Aragam KG (2018). Genome-wide polygenic scores for common diseases identify individuals with risk equivalent to monogenic mutations. Nat Genet.

[24] Marston NA, Garfinkel AC, Kamanu FK (2023). A polygenic risk score predicts atrial fibrillation in cardiovascular disease. Eur Heart J.

[25] Mars N, Koskela JT, Ripatti P (2020). Polygenic and clinical risk scores and their impact on age at onset and prediction of cardiometabolic diseases and common cancers. Nat Med.

[26] Shoemaker MB, Husser D, Roselli C (2020). Genetic susceptibility for atrial fibrillation in patients undergoing atrial fibrillation ablation. Circ Arrhythm Electrophysiol.

[27] Kany S, Al-Taie C, Roselli C (2023). Association of genetic risk and outcomes in patients with atrial fibrillation: interactions with early rhythm control in the EAST-AFNET4 trial. Cardiovasc Res.

[28] Kertai MD, Mosley JD, He J (2021). Predictive accuracy of a polygenic risk score for postoperative atrial fibrillation after cardiac surgery. Circ Genom Precis Med.

[29] https://academic.oup.com/europace/article/24/8/1307/6562982?login=true

[30] Richards S, Aziz N, Bale S (2015). Standards and guidelines for the interpretation of sequence variants: a joint consensus recommendation of the American College of Medical Genetics and Genomics and the Association for Molecular Pathology. Genet Med.

[31] Yoneda ZT, Anderson KC, Quintana JA (2021). Early-onset atrial fibrillation and the prevalence of rare variants in cardiomyopathy and arrhythmia genes. JAMA Cardiol.

[32] Yoneda ZT, Anderson KC, Ye F (2022). Mortality among patients with early-onset atrial fibrillation and rare variants in cardiomyopathy and arrhythmia genes. JAMA Cardiol.

[33] Goodyer WR, Dunn K, Caleshu C (2019). Broad genetic testing in a clinical setting uncovers a high prevalence of titin loss-of-function variants in very early onset atrial fibrillation. Circ Genom Precis Med.

[34] Verdonschot JAJ, Hazebroek MR, Krapels IPC (2020). Implications of Genetic Testing in Dilated Cardiomyopathy. Circ Genom Precis Med.

[35] Stroeks SLVM, Lunde IG, Hellebrekers DMEI (2023). Prevalence and clinical consequences of multiple pathogenic variants in dilated cardiomyopathy. Circ Genom Precis Med.

[36] Miller DT, Lee K, Abul-Husn NS (2023). ACMG SF v3.2 list for reporting of secondary findings in clinical exome and genome sequencing: a policy statement of the American College of Medical Genetics and Genomics (ACMG). Genet Med.

[37] Miller DT, Lee K, Gordon AS (2021). Recommendations for reporting of secondary findings in clinical exome and genome sequencing, 2021 update: a policy statement of the American College of Medical Genetics and Genomics (ACMG). Genet Med.

[38] Alhassani S, Deif B, Conacher S (2018). A large familial pathogenic plakophilin-2 gene (PKP2) deletion manifesting with sudden cardiac death and lone atrial fibrillation: evidence for alternating atrial and ventricular phenotypes. Heart Rythm Case Rep.

[39] Darbar D, Kannankeril PJ, Donahue BS (2008). Cardiac sodium channel (SCN5A) variants associated with atrial fibrillation. Circulation.

[40] Jurgens SJ, Choi SH, Haggerty CM (2022). Abstract 13496: Sequencing in over 50,000 cases identifies coding and structural variation underlying atrial fibrillation risk. Circulation.

